# Supplementary material for: Complexome profiling on the Chlamydomonas lpa2 mutant reveals insights into PSII biogenesis and new PSII associated proteins
Source: J Exp Bot. 2021 Aug 26;73(1):245–62. doi: 10.1093/jxb/erab390 (PMC8730698; doi:10.1093/jxb/erab390)
Supplement: erab390_suppl_Supplementary_Dataset_S1 [file erab390_suppl_supplementary_dataset_s1.zip › Supplemental Dataset 1 - Excel List and all profiles/plots/GCST_Cre03.g193750.html]

Trivial name: GCST  
  
Euclidean distance: 30220.91  
Mean Intensity (WT): 4498.09  
Mean Intensity (Mut): 2808.19  
Distance: 6.72  
  
MapMan: amino acid metabolism.degradation.serine-glycine-cysteine group.glycine;PS.photorespiration.glycine cleavage.T subunit  
  
p value of intensity sums Welch test: 0.5049
